# Gene- and stem cell-based therapeutics for cartilage regeneration and repair

**DOI:** 10.1186/s13287-015-0058-5

**Published:** 2015-04-15

**Authors:** Ying Tang, Bing Wang

**Affiliations:** Department of Orthopaedic Surgery, University of Pittsburgh School of Medicine, 450 Technology Drive, Suite 216, Pittsburgh, PA 15219 USA

## Abstract

Cell-based regeneration of damaged or diseased articular cartilage still faces significant clinical challenge due to inadequate environmental regulation of stem cell proliferation and chondrogenic differentiation. The role of insulin-like growth factor in critical steps of human bone marrow-derived mesenchymal stem cell chondrogenesis has potential in optimizing the therapeutic use of mesenchymal stem cells in cartilage disorders. In addition to the previously described benefits of recombinant adeno-associated viral vector for *in vivo* gene therapy, demonstrated by Frisch and colleagues, such vector is also a safe and efficient delivery system for the genetic modification of human bone marrow-derived mesenchymal stem cells via *ex vivo* insulin-like growth factor 1 gene transfer, so that implanted mesenchymal stem cells continuously release a therapeutic level of insulin-like growth factor 1 to achieve sustained mesenchymal stem cell chondrogenesis for cartilage regeneration.

## Introduction

Currently, no effective treatment for articular cartilage injuries and disorders has been developed to rescue the impaired cartilage or prevent damage progression or both. Progressive degeneration of the articular cartilage inevitably leads to pain, swelling, and stiffness of the joint. Although current surgical approaches can temporarily alleviate the course of injury or disease, it is often challenged by the poor intrinsic healing potential of articular cartilage. Stem cell-based tissue engineering for cartilage regeneration is a promising alternative pathway in the regeneration of damaged or diseased articular cartilage tissues or both; nevertheless, it needs to be optimized to enhance cell proliferation and chondrogenic differentiation. To induce chondrogenesis of human bone marrow-derived mesenchymal stem cells (hMSCs), chondroinductive biofactors, such as insulin-like growth factor 1 (IGF-1), are often used. However, these proteins’ short half-lives and rapid clearance by the bloodstream, shown in experimental animals, limit their utility. So there is a critical need for the development of a safe and effective delivery system to stably deliver *IGF-1* to achieve a sustained, long-term effect on chondrogenesis of hMSCs. In a recent issue of *Stem Cell Research & Therapy*, Frisch and colleagues [1] demonstrate that the *ex vivo* gene transfer of *IGF-1* to stem cells through a recombinant adeno-associated viral (rAAV) vector system, prior to their seeding onto biomaterial or implantation or both, is effective for sustained production of IGF-1. The IGF-1 expressed from hMSCs will self-induce chondrogenic differentiation and promote cartilage regeneration. For safety concerns, a tight regulation of the rAAV vector gene delivery system needs to be used.

The regeneration of damaged or diseased articular cartilage tissues remains a significant clinical challenge. Current medical treatments are effective at reducing pain but ineffective at reversing the course of musculoskeletal degeneration often due to the low cellularity and vascularity in these tissues. As a result, degenerative cartilage tissues do not heal properly. Conventional therapies for the treatment of the latter conditions consist of treating the damaged tissues with anti-inflammatory drugs or using replacement therapies with synthetic materials or cartilage grafting. In traditional drug therapy, the desired protein or growth factor is delivered directly to the site of injury by injection; however, the material is quickly degraded and eliminated by the body such that the therapeutic concentration of the required agent is difficult to maintain at the site of injury. Although surgeries can temporarily arrest the course of disease and injury, replacement surgeries frequently fail and ultimately result in increased cartilage tissue damage. Therefore, despite the variety of surgical techniques, cell-based cartilage tissue engineering is an attractive alternative approach in the reconstruction of articular cartilage. More recently, Frisch and colleagues demonstrated that the combination of gene and cell therapies could provide a novel treatment for articular cartilage degenerative diseases in the not-too-distant future [1].

Cartilage tissue engineering using hMSCs has demonstrated the potential to enhance cartilage healing [2]. Current challenges in cell-based cartilage tissue engineering are concerned primarily with unsuitable environmental regulation of hMSC attachment, cell proliferation, and chondrogenic differentiation [3]. Thus, a major requirement in hMSC-based cartilage tissue engineering is to promote chondrogenic differentiation of cells, which requires the continuous introduction of chondroinductive biofactors, such as pro-anabolic IGF-1. The biological role of IGF-I in proliferation, chondrogenic condensation, apoptosis, and differentiation of hMSCs into chondrocytes has been investigated in the last decade [4]. Most of the recent approaches, involving the incorporation of IGF-1 protein into damaged tissue or culturing hMSCs prior to their implantation, have limited success because of the short functional half-life of IGF-1, rapid clearance of IGF-1 by the bloodstream, and the time-consuming and cost-prohibitive additional cell-culture steps. Therefore, intra-articular injection of IGF-1 to stimulate cartilage repair may not be the most practical therapy [5]. There is an urgent need for the development of an effective delivery system for *IGF-1* to achieve a sustained, long-term chondrogenic effect on hMSCs.

rAAV vectors are manufactured from non-pathogenic and non-replicative parvoviruses [6]. They have the distinctly advantageous biological property of being capable of infecting a wide range of host cell types, including dividing and non-dividing cells, which makes rAAV vector a particularly good vector for delivering growth factors for treating injured tissues. rAAV vector-based gene therapy represents one of the most promising approaches to aid in the repair and regeneration of bone, ligament, tendon, and joint capsules [7]. The benefits of using rAAV vector include superior long-term gene transfer efficiency, the absence of an immune response to the virus, and the lack of toxicity and mutagenesis, which are often associated with other viral vectors. The efficiency, safety, and convenience associated with rAAV vector-mediated gene therapy have led to the initiation of phase I and II clinical trials in humans [8].

First, the insight from this study is the use of rAAV vector as an *IGF-1* delivery system to sustain stable expression of IGF-1 in undifferentiated and chondrogenically induced hMSCs up to 21 days. Enhanced proliferative, biosynthetic, and chondrogenic activities were observed in IGF-transduced hMSCs, demonstrated by a large amount of simultaneously increased expression of separate genes without interference, such as *SOX9, COL2A1, COL1A1, COL10A1, MMP13, RUNX2, ALP*, and beta-catenin, in chondrogenically induced cultures of hMSCs. In the future, the *in vivo* applicability of rAAV vector-*IGF-1* transduced hMSCs *in vitro* should be assessed by transplanting such IGF-1-positive hMSCs into damaged or diseased cartilage tissue. Furthermore, the implanted genetically modified hMSCs at the site of injury may continuously release high therapeutic levels of IGF-1, which not only enhance chondrogenic differentiation of implanted hMSCs themselves but also have a variety of physiological effects on the different types of cells in the body. As a result, implantation of rAAV vector-based *IGF-1* genetically modified hMSCs will increase vascularity and the influx of reparative cells via chemo-attraction and improve the intrinsic capacity of the damaged cartilage tissue to heal itself.

Second, this study demonstrates that overexpression of IGF-1 in hMSCs may also increase the expression of hypertrophic and osteogenic markers. Owing to multiple biological properties of IGF-1, the genetically modified rAAV vector-*IGF-1* hMSCs also showed osteogenic and adipogenic differentiation potentials under proper induction conditions *in vitro*. Therefore, as Payne and colleagues mentioned previously, highly regulated delivery of *IGF-1* using an inducible rAAV vector system will be critical to achieve an optimized hMSC-based cell therapy to treat articular cartilage defects *in vivo* in the future [9].

In conclusion, Frisch and colleagues have practiced rAAV vector-mediated *ex vivo* gene transfer of IGF-1 in hMSCs as a therapeutic option to treat cartilage degenerative diseases. Besides the selection of an optimal controllable rAAV vector delivery system, the use of an appropriate rAAV vector serotype or a capsid-modified rAAV vector [10] can achieve highly efficient and sustainable *IGF-1* gene transfer to promote chondrogenic potential of hMSCs for cartilage regeneration and further can offset losses of rAAV vector viral genome dilution during hMSC proliferation because rAAV vector genome persists primarily as speisomal chromatin in the nucleus of hMSCs [11].

## Abbreviations

hMSC: Human (bone marrow-derived) mesenchymal stem cell
IGF-1: Insulin-like growth factor 1
rAAV: Recombinant adeno-associated viral
